# Effect of fatigue and gender on kinematics and ground reaction forces variables in recreational runners

**DOI:** 10.7717/peerj.4489

**Published:** 2018-03-20

**Authors:** Bruno Bazuelo-Ruiz, Juan V. Durá-Gil, Nicolás Palomares, Enrique Medina, Salvador Llana-Belloch

**Affiliations:** 1 Department of Physical Education and Sports, University of Valencia, Valencia, Spain; 2 Instituto de Biomecánica de Valencia, Valencia, Spain

**Keywords:** Biomechanics, Injury risk, Running economy, Running performance

## Abstract

The presence of fatigue has been shown to modify running biomechanics. Overall in terms of gender, women are at lower risk than men for sustaining running-related injuries, although it depends on the factors taken into account. One possible reason for these differences in the injury rate and location might be the dissimilar running patterns between men and women. The purpose of this study was to determine the effect of fatigue and gender on the kinematic and ground reaction forces (GRF) parameters in recreational runners. Fifty-seven participants (28 males and 29 females) had kinematic and GRF variables measured while running at speed of 3.3 m s^−1^ before and after a fatigue test protocol. The fatigue protocol included (1) a running Course-Navette test, (2) running up and down a flight of stairs for 5 min, and (3) performance of alternating jumps on a step (five sets of 1 minute each with 30 resting seconds between the sets). Fatigue decreased dorsiflexion (14.24 ± 4.98° in pre-fatigue and 12.65 ± 6.21° in fatigue condition, *p* < 0.05) at foot strike phase in females, and plantar flexion (−19.23 ± 4.12° in pre-fatigue and −18.26 ± 5.31° in fatigue condition, *p* < 0.05) at toe-off phase in males. These changes led to a decreased loading rate (88.14 ± 25.82 BW/s in pre-fatigue and 83.97 ± 18.83 BW/s in fatigue condition, *p* < 0.05) and the impact peak in females (1.95 ± 0.31 BW in pre-fatigue and 1.90 ± 0.31 BW in fatigue condition, *p* < 0.05), and higher peak propulsive forces in males (−0.26 ± 0.04 BW in pre-fatigue and −0.27 ± 0.05 BW in fatigue condition, *p* < 0.05) in the fatigue condition. It seems that better responses to impact under a fatigue condition are observed among women. Further studies should confirm whether these changes represent a strategy to optimize shock attenuation, prevent running injuries and improve running economy.

## Introduction

Despite of the health benefits provided by running, overuse injuries are a matter of concern, especially in less experienced runners. Injury rates range between 18.2 and 92.4% among runners (Saragiotto et al., 2014). Overall, women are at lower risk than men for sustaining running-related injuries, although it depends on the factors taken into account (Worp et al., 2015). For instance, males have a higher prevalence for certain injuries, such as patellar tendinopathy or adductor strain, while females suffer patellar femoral pain syndrome and sacroiliac injuries at a higher rate (Watson & DiMartino, 1987; Taunton et al., 2002). One possible reason for the differences in the injury rate and location might be the different running patterns employed by men and women. Some authors have noted that females exhibited greater peak hip internal rotation and adduction during walking and running compared with males (Chumanov, Wall-Scheffler & Heiderscheit, 2008). Besides, while Chao et al. (1983) found higher values of impact peak force in males than in females during walking, Keller et al. (1996) reported no gender-related differences in ground reaction force (GRF) variables during walking and running.

The presence of fatigue has been shown to modify running biomechanics (Derrick, Dereu & McLean, 2002). However, whether the management of fatigue is appropriate, and the workload and rest time are optimal, fatigue could generate positive adaptations that would be beneficial for the athlete (Fry & Kraemer, 1997; Kreher & Schwartz, 2012). The sense of effort is associated with neural or neuromuscular fatigue (Noakes, 2004) and the changes in perceived exertion that result from multiple afferent signals may allow exercise performance to be precisely regulated such that a task can be completed within the biomechanical and metabolic limits of the body (Hampson et al., 2001). Some authors have indicated that although the movement pattern is altered with muscular fatigue, the goal of the task is preserved (Gates & Dingwell, 2008). With respect to the kinematic parameters, previous studies have shown that knee and ankle joint angles at foot strike phase are important factors for joint stability, while joint kinematics at toe-off are important determinants of running performance (Gazendam & Hof, 2007). However, a significant controversy exists about the fatigue effects on the biomechanics of running. While Nicol, Komi & Marconnet (1991) did not find any changes after a marathon, Clansey et al. (2012), Derrick, Dereu & McLean (2002) and Dutto et al. (1997) noted that the knee and ankle were more flexed in the contact phase when runners were fatigued. Flexing the knee and ankle may shift the shock-attenuating responsibilities away from passive biological tissue toward active muscular contraction (Edwards, Derrick & Hamill, 2012). Hence, the study of lower limb kinematics in the stance phase is of interest because of its direct relationship with other biomechanical parameters, i.e., ground reaction forces (GRF) and impact shock. Moreover, such a study would improve the existing knowledge about the injury risk and the enhancement of running performance.

Other authors have revealed that vertical ground reaction force (VGRF) values reduced with fatigue (Nicol, Komi & Marconnet, 1991; Christina, White & Gilchrist, 2001; Gerlach et al., 2005; Morin, Samozino & Millet, 2011), while others have concluded higher values when runners were fatigued (Dickinson, Cook & Leinhardt, 1985; Christina, White & Gilchrist, 2001). All these scientific articles have analysed different biomechanical parameters in males (Dickinson, Cook & Leinhardt, 1985; Morin, Samozino & Millet, 2011; Clansey et al., 2012) and females (Christina, White & Gilchrist, 2001; Gerlach et al., 2005), and have usually reported the same conclusions for both populations. Just one publication (Dutto et al., 1997) compared 12 males and 13 females, though these were elite rather than recreational or competitive runners.

Therefore, the purpose of this study was to determine the effect of fatigue and gender on kinematic and GRF parameters in recreational runners. We hypothesized that males would exhibit higher knee flexion in the foot strike phase while no significant differences would exist in the ankle flexion angle. Moreover, it was also hypothesized that females would modify their knee and ankle angles in a higher flexion when fatigued in all the running phases analysed. Furthermore, it was speculated that similar values would be found in the GRF normalized to body weight in both females and males with and without fatigue, while the loading rate and first impact peak would be reduced in the presence of fatigue, whereas the maximum value of the VGRF would not suffer any change at constant running speed. Finally, with regard to the antero-posterior GRF, it was proposed that the braking force in the foot strike phase would increase and the propulsive force would be reduced.

## Materials & Methods

### Participants

Fifty-seven recreational and heel strike runners (28 males and 27 females) were recruited for this investigation. The physical characteristics of the participants in this study are presented in Table 1. The inclusion criteria of the sample required that the participants were 18 to 55 years old, running as main physical activity practice performed two to three days a week with a weekly volumne of 20 to 40 km. Participants were excluded if they had any cardiovascular disease or anatomic alterations that could influence their running pattern. They were injury-free six months prior to testing. In addition, they refrained from eating for at least two hours before testing and did not perform vigorous exercise in the 24 h prior to the test. All participants gave their written informed consent prior to participation in the study.

**Table 1:** Physical characteristics of the participants (recreational runners) for the data analysis.

	Age (years)	Body mass (kg)	Height (m)	BMI (kg/m^2^)
Male (*n* = 28)	34 ± 10	72.6 ± 6.8	1.76 ± 0.06	23.5 ± 1.9
Female (*n* = 29)	32 ± 8	59.3 ± 8.1	1.66 ± 0.04	21.6 ± 2.4
Total (*n* = 57)	33 ± 9	65.8 ± 10.0	1.71 ± 0.07	22.5 ± 2.3

**Notes.**

BMI, body mass index; Ranges of BMI: Underweight = 16 to 18.5; Normal = 18.5 to 25; Overweight = 25 to 30; Obese = 30 to 35.

### Procedures

Kinematic and GRF variables were measured under two conditions: before and after a fatigue protocol. For data collection purposes, subjects ran in the laboratory on a 15 m long runway equipped with a flush-to-the-floor mounted force plate (Dinascan/IBV, Valencia, Spain) with a sampling frequency of 1,000 Hz. The force plate was synchronized to a 3D motion capture analysis system (Kinescan/IBV, Valencia, Spain) with 10 cameras operating at a sampling frequency of 250 Hz.

A successful trial was considered when the runner contacted the force platform with the entire right foot and the running speed was 3.3 m s^−1^ (±5%). The running speed was monitored by two sets of photocells positioned on either side of the force plate and connected to a chronometer. Five successful trials were recorded for each subject and condition.

All participants wore the same neutral running shoes (Kelme Gravity MC, Kelme, Spain) of appropriate size. The fatigue protocol consisted three consecutive tasks: (1) a running Course-Navette test (Léger et al., 1988), (2) running up and down a flight of stairs for 5 min, and (3) alternative jumps on a step (five sets of 1 min each with 30 resting seconds between the sets). Between each task, the rest time was about 1 min. The subjects were familiarized with the fatigue protocol because they had been previously in the laboratory for another research project that performed the same fatigue protocol. The protocol was performed once by each subject and concluded when the participants reached 90% of their theoretical maximal heart rate (Tanaka, Monahan & Seals, 2001) and when a score of 18 was indicated by the participants on the visual Borg Scale RPE 6-20 (Borg, 1982). The scale was presented to the runners every three minutes. The study protocol adhered to the tenets of the Declaration of Helsinki and ethical approval was given by the local university Ethics Committee with the number 229.

### Data analysis

**Figure 1:**
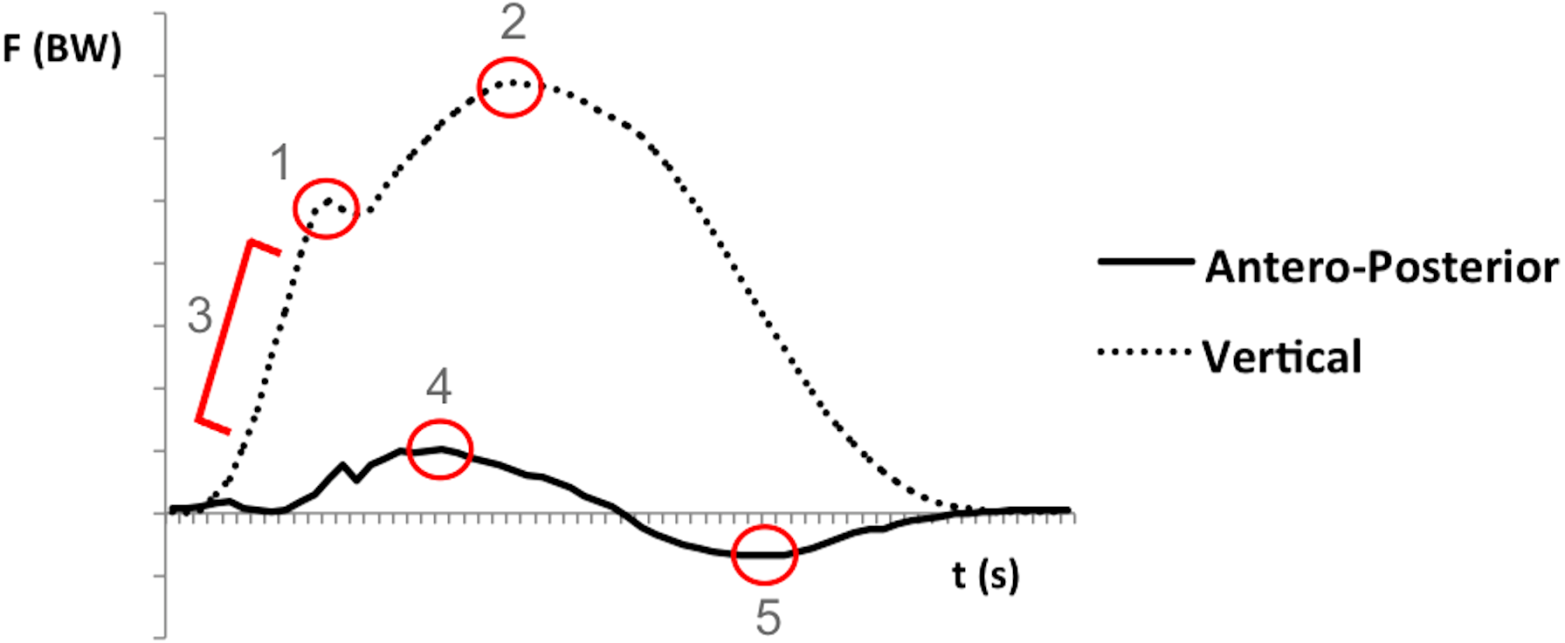
Representative ground reaction forces (vertical and antero-posterior components) from the stance phase of one running stride. Force was normalized to body weight (BW). (1) Impact peak force; (2) active peak force; (3) loading rate; (4) peak braking force; (5) peak propulsive force. Dotted line for vertical force and continuous line for antero-posterior force.

All data were processed and extracted using a custom program written in Matlab (MATLAB, The Mathworks, Inc, Natick, MA, USA). From the vertical component of GRF, the impact peak force, the loading rate and the active peak force were determined. The loading rate was the rate of change of force between 20 and 80% from the moment of foot strike and the impact peak force. The braking and propulsive forces were obtained from the antero-posterior component. Force data were normalized to body weight (Fig. 1).

The knee and ankle joint angles were determined by a biomechanical model according to the International Society of Biomechanics’ indications (Wu et al., 2002). All curves were smoothed using a B-splines base (Medina et al., 2013) and time scales were linearly adjusted in order to express the evolution of the movement as a percentage of the stance phase. From each curve, the angle value was determined at three phases: foot strike (FS), full contact (FC) of the foot with the ground and just before toe-off (TO). The FS was determined when the VGRF was >10 N (Wright et al., 2013), the FC when the maximum dorsiflexion angle was reached, and the TO when the GRF were <10 N. Full knee extension was defined as an angle of 0°, corresponding to the reference anatomic position.

### Statistical analysis

Mean, standard deviation (SD) and 95% confidence intervals were calculated for all variables. The Shapiro–Wilk test was performed on all measured variables to determine their distribution. A two-way mixed model ANOVA (with gender and fatigue condition as factors) was used. All statistical analyses were conducted using SPSS Statistics v.20 (IBM, Chicago, IL, USA). The statistical significance was defined as *p* ≤ 0.05.

## Results

Gender and fatigue effects were observed in the GRF and kinematic data (Table 2). Regarding the gender effect on kinematics, all variables analysed were significantly different for males and females in both the pre-fatigue and fatigue conditions. Higher dorsiflexion and knee angles in both pre-fatigue and fatigue conditions were observed in females compared with males, except for a lower plantar flexion in the TO phase for males. From GRF analysis, females exhibited a significantly higher loading rate but a lower active peak force for the GRF in both the pre-fatigue and fatigue conditions when compared with males. However, the impact peak force and the loading rate were significantly reduced in the group of females (analysed independently) when they were in a fatigued state.

**Table 2:** Gender and fatigue effects on the kinematic and GRF parameters (males *n* = 28; females *n* = 29; total sample *n* = 57 recreational runners).

	Pre-fatigue			Fatigue		
	Males	Females	95% CI	Males	Females	95% CI
**GRF (BW)**						
Impact peak force	1.91 ± 0.31	1.95 ± 0.31^d^	−0.12 to 0.03	1.88 ± 0.32	1.90 ± 0.31^d^	−0.09 to 0.06
Active peak force	2.59 ± 0.22^a^	2.47 ± 0.19^a^	0.06 to 0.16	2.61 ± 0.20^b^	2.46 ± 0.19^b^	0.11 to 0.19
Loading rate (BW/s)	75.02 ± 17.48^a^	88.14 ± 25.82^a^^,^^d^	−18.29 to −7.94	74.26 ± 19.90^b^	83.97 ± 18.83^b^^,^^d^	−14.24 to −5.16
Peak braking force	0.36 ± 0.09	0.38 ± 0.08	−0.03 to 0.01	0.36 ± 0.09	0.38 ± 0.07	−0.03 to −0.003
Peak propulsive force	−0.26 ± 0.04^a^^,^^c^	−0.28 ± 0.04^a^	0.004 to 0.02	−0.27 ± 0.05^c^	−0.28 ± 0.04	−0.01 to −0.02
**KINEMATICS**						
**Knee angles** (°)						
FS	7.37 ± 5.18^a^	12.96 ± 5.71^a^	−7.17 to −4.00	7.42 ± 4.57^b^	13.11 ± 5.97^b^	−7.22 to −4.14
TO	−12.04 ± 6.33^a^	−13.97 ± 4.70^a^	−3.56 to −0.29	−11.52 ± 5.59^b^	−14.56 ± 6.49^b^	−4.79 to −1.28
FC	36.89 ± 6.38^a^	41.48 ± 4.54^a^	−6.20 to −2.98	36.57 ± 5.29^b^	41.39 ± 5.53^b^	−6.38 to −3.26
**Ankle angles** (°)						
FS	9.97 ± 4.93^a^	14.24 ± 4.98^a^^,^^d^	−5.77 to −2.78	10.39 ± 4.26^b^	12.65 ± 6.21^b^^,^^d^	−3.87 to −0.63
TO	−19.23 ± 4.12^a^^,^^c^	−13.96 ± 3.28^a^	−6.36 to −4.17	−18.26 ± 5.31^b^^,^^c^	−13.57 ± 4.60^b^	−6.14 to −3.23
FC	19.71 ± 3.19^a^	23.07 ± 3.20^a^	−4.26 to −2.45	20.15 ± 3.80^b^	22.67 ± 4.46^b^	−3.69 to −1.35

**Notes.**

GRF Ground Reaction Forces BW Body Weight FS Foot Strike TO Toe-Off FC Full Contact

a Significant differences between males and females in the pre-fatigue condition.

b Significant differences between males and females in the fatigue condition.

c Significant differences between the pre-fatigue and fatigue conditions in males.

d Significant differences between the pre-fatigue and fatigue conditions in females.

The statistical significance was defined as *p* ≤ 0.05.

With respect to the fatigue condition, the peak propulsive force in females was higher in the pre-fatigue condition compared with males, while only in males a fatigue effect was observed for this parameter, being higher in the fatigue condition. From kinematic analysis, fatigue only affected the FS ankle angle in females, being less flexed in the fatigue condition. Moreover, in the TO phase, the ankle was less extended in males in the fatigue condition. The knee angle was not affected by neither gender nor fatigue effects.

## Discussion

The aim of this study was to determine the effect of fatigue and gender on kinematic and GRF parameters in recreational runners. The main results of the present work are that the loading rate and the impact peak of the VGRF were lower in the group of females when they were fatigued, and the maximum propulsive force was higher in the group of males in the fatigue condition. When comparing females and males, the loading rate was higher in females in both conditions. Furthermore, regarding kinematics, the dorsiflexion angle at the FS phase in females was significantly reduced in the fatigue condition, and the ankle angle at TO phase showed less plantar flexion in males also in the fatigue condition.

The nature of running involves a storage restitution of elastic energy within the musculo-tendinous structures of the lower limbs during the stretch-shortening cycle. The runner’s legs alternately store and release elastic energy, which has been proposed as a spring-mass model (Blickhan, 1989; Geyer, Seyfarth & Blickhan, 2005), although other models have been proposed more recently to estimate leg stiffness (Coleman et al., 2012). Accordingly, the knee joint plays a critical role in the body’s ability to absorb shock and force dissipation during ground contact (Zhang, Bates & Dufek, 2000; Hargrave et al., 2003). Lower knee flexion during landing may lead to a reduced time for shock attenuation, which has been associated with knee joint injuries (Hargrave et al., 2003; Jeffrey & Aspden, 2006; Coventry et al., 2006), and a weaker knee joint power (Devita & Skelly, 1992). Moreover, a very recent study reported that kinematic changes of the knee may be interpreted as shifting the stress applied to the cartilage and menisci (Slater et al., 2018). In the present study, no changes were found in the knee angle when runners were fatigued. On this basis, it could be understood that the knee flexion angle does not depend on the fatigue protocol, but on the running speed. Some studies reported this state, indicating that the leg spring stiffness is influenced mostly by changes in stiffness at the knee joint at higher speeds (Arampatzis, Brüggemann & Metzler, 1999; Schache et al., 2011).

On the other hand, it has been proposed that fatigue could result in a reduction of the protection capacity of the fatigued muscles (Mizrahi et al., 2000). In this sense, although our results did not show a shift of the knee angle with fatigue, females experimented a modification in ankle angle (lower dorsiflexion). This shift led to a lower loading rate and the impact peak of the VGRF in females. It could be understood as a shift in the biomechanical pattern in females in order to optimize shock attenuation, prevent running injuries or improve running economy (RE) when they were fatigued. Nevertheless, this hypothesis should be contrasted in further studies.

The ankle joint also has an important role in shock absorption when the foot touches the ground. A small modification in the ankle dorsiflexion could directly affect GRF (Devita & Skelly, 1992; Kovács et al., 1999; Self & Paine, 2001). Gerritsen, Van den Bogert & Nigg (1995) showed that a higher ankle dorsiflexion resulted in an increase in the first peak force and the loading rate. Other authors have indicated that a runner’s ankle joint experiences a lower dorsiflexion when fatigued (Dutto et al., 1997). In this respect, we found that the ankle dorsal flexion was reduced in females than in males after the fatigue protocol. This is in agreement with the findings by Kellis, Zafeiridis & Amiridis (2011) and Christina, White & Gilchrist (2001) who found that the ankle dorsal flexion at FS was 3.2° lower when running after a fatigue protocol for the dorsiflexors. In our study, this fact led to a decrease in the first peak force and the loading rate in females. This association was not observed in males. In contrast, fatigued males exhibited lower plantar flexion in the TO phase and a higher peak propulsive force. Regarding the TO phase, the ankle angle has also been investigated in the literature due to its association with the RE. Moore, Jones & Dixon (2012) found that the plantarflexion in the TO phase was lower and the peak propulsive force was higher after a 10-week running training program at the same time as the RE was improved. Similar changes in the plantarflexion were detected in runners with better RE (Williams & Cavanagh, 1987; Moore, Jones & Dixon, 2014). Although oxygen consumption was not measured in this study, male runners might modify their running pattern seeking a better RE when in a fatigued state. With respect to gender, many authors recruited males and females for their studies finding the same conclusions for both groups. However, several differences exist in their biomechanical and muscle activation patterns. Our findings showed higher knee and ankle flexion angles in females in all running phases analysed. The results of the study by Gehring, Melnyk & Gollhofer (2009) are in agreement with our outcomes, giving higher maximum knee angles in females. In contrast, Malinzak et al. (2001) noted a reduction of 8° in knee flexion in females compared with males. Previous studies found greater peak hip internal rotation (2.4 ± 3.3° in males versus 6.2 ± 4.3° in females) and adduction (8.1 ± 2.2° in males versus 11.0 ± 3.0° in females), as well as gluteus maximus activity in females (Chumanov, Wall-Scheffler & Heiderscheit, 2008). To the best of our knowledge, only one study evaluated the fatigue effect in the GRF in females (Gerlach et al., 2005) and found a decreased first impact peak and loading rates during fatigue. The present work provided the same result in this regard. However, it is important to consider that our fatigue protocol involves running but it was not a specific running protocol. A fatigue protocol that involves running may results in different outcomes due to inherent patterns of muscle activation as well as energy depletion. From a practical point of view, our results suggest that care should be taken into account for gender when analyzing groups of male and females recreational runners.

## Conclusions

In summary, gender and fatigue effects were observed for GRF and kinematics. Runners adapt their running pattern with fatigue, resulting in decreased dorsiflexion in the FS phase in females and less plantarflexed ankle in the TO phase in males. These changes led to decreased loading rates and first impact peak in females and higher peak propulsive forces in males. Further studies should confirm whether these changes represent a strategy to optimize shock attenuation, prevent running injuries and improve RE.

##  Supplemental Information

10.7717/peerj.4489/supp-1 Supplemental Information 1 Ankle kinematics

10.7717/peerj.4489/supp-2 Supplemental Information 2 Knee kinematics

10.7717/peerj.4489/supp-3 Supplemental Information 3 Ground reaction forces

## Additional Information and Declarations

### Data Availability

The following information was supplied regarding data availability: The raw data are provided in the Supplemental Files.
